# Intramolecular electron-induced proton transfer and its correlation with excited-state intramolecular proton transfer

**DOI:** 10.1038/s41467-019-09154-5

**Published:** 2019-03-12

**Authors:** Wei Wang, Mary Marshall, Evan Collins, Sara Marquez, Chaonan Mu, Kit H. Bowen, Xinxing Zhang

**Affiliations:** 1 Key Laboratory of Advanced Energy Materials Chemistry (Ministry of Education), College of Chemistry, Nankai University, 300071 Tianjin, China; 2 Department of Chemistry, Johns Hopkins University, Baltimore, MD 21218 USA

## Abstract

Electron-induced proton transfer depicts the proton motion coupled with the attachment of a low-energy electron to a molecule, which helps to understand copious fundamental chemical processes. Intramolecular electron-induced proton transfer is a similar process that occurs within a single molecule. To date, there is only one known intramolecular example, to the best of our knowledge. By studying the 10-hydroxybenzo[h]quinoline and 8-hydroxyquinoline molecules using anion photoelectron spectroscopy and density functional theory, and by theoretical screening of six other molecules, here we show the intramolecular electron-induced proton transfer capability of a long list of molecules that meanwhile have the excited-state intramolecular proton transfer property. Careful examination of the intrinsic electronic signatures of these molecules reveals that these two distinct processes should occur to the same category of molecules. Intramolecular electron-induced proton transfer could have potential applications such as molecular devices that are responsive to electrons or current.

## Abstract (web summary)

Intramolecular electron-induced proton transfer (iEIPT) describes proton motion coupled with the attachment of an electron within a molecule. Here, the authors build a “search engine” for iEIPT-capable molecules that also have the excited-state intramolecular proton transfer property.

## Introduction

Electron-induced proton transfer (EIPT) describes the proton motion coupled with the attachment of a low-energy electron to a molecule. It widely occurs in nature and has been observed in many different systems^1–20^. EIPT helps to understand copious fundamental chemical processes such as acid-base chemistry: it is known that in some simple acid-base reactions, the proton transfer from one acid molecule (e.g. HCl, HBr, HI and H_2_O) to another base molecule (e.g. NH_3_ and pyridine) cannot occur. The attachment of an electron otherwise facilitates these reactions due to the formation of a transient dipole bound anionic state^1,2^ or a unstable valence bound anionic state^3^. EIPT is also considered to be accountable for the damage of DNA in cancer radiotherapy^4,5^. Secondary electrons generated by high-energy photons attach to DNA molecules and induce EIPT between the double strands. As a result, normal and cancerous cells are killed indistinguishably. In order to understand DNA damage by low-energy electrons at the molecular level, gas phase isolated model systems using DNA subunits such as nucleobases and other biologically relevant molecules have been studied in anion beams spectrometrically and spectroscopically. These model systems include EIPT between guanine and cytosine^6^, uracil and alanine^7^, uracil and glycine^8^, thymine and glycine^9^, uracil dimer^10^, thymine dimer^10^, adenine and 9-methyladenine^11^, adenine and thymine^12^, 9-methyladenine and 1-methylthymine^13^, formic acid and adenine^13^, formic acid and 1-methylcytosine^14^, formic acid and thymine^15^, formic acid and uracil^15^, uracil and alcohols^16^, uracil and H_2_S/H_2_Se/HCN^17,18^. Conclusively, EIPT is vital in understanding many fundamental chemical and biological processes in nature.

All of the above-mentioned EIPT examples, nevertheless, occur between at least two molecules, i.e. they are intermolecular EIPT. Here we show some other molecules with their local structures satisfying certain conditions could undergo intramolecular EIPT (iEIPT). Reactions that occur within one molecule, which is the smallest environment that a chemical reaction could have, are interesting for its own sake. It can be inferred that a certain molecule should satisfy at least three criteria to undergo iEIPT: (1) it should have one functional group as the proton donor (such as −OH or −COOH) and one functional group as the proton receptor (nucleophiles such as lone pair); (2) these two functional groups should be in close proximity and exhibit a pre-existing hydrogen bond; (3) this molecule should be able to electronically accommodate one excess electron in a manner of a π* antibonding orbital. Historically, iEIPT was only observed in the acetoacetic acid anion^20^ whose neutral counterpart fulfills the above three criteria. To systematically search for molecules of this kind, we examined another class of molecules having the ability of excited-state intramolecular proton transfer (ESIPT)^21–42^. Numerous experimental and theoretical efforts have been paid to ESIPT owing to its applications^33–38^ in molecular probes, luminescent materials, molecular logic gates, etc. It is not surprising that all of the ESIPT molecules fulfill the above three criteria of iEIPT. With the help from the well-documented ESIPT molecules, much more iEIPT molecules could be potentially discovered. Nevertheless, ESIPT involves the excited states of neutral molecules while EIPT deals with anionic systems; they are very different in nature at the first glance. Therefore, even if these two categories of proton transfer happen to occur in the same molecules, the intrinsic property that facilitates this “coincidence” is yet to be discovered.

Here, we report the iEIPT capability of 10-hydroxybenzo[h]quinoline (HBQ) and 8-hydroxyquinoline (HQ), both of which have been shown to display ESIPT properties^24–32^, especially the former, HBQ, was extensively studied both experimentally and theoretically^24–31^. Here the iEIPT of these two molecules are studied by mass spectrometry and photoelectron spectroscopy, and the experimental results are then validated by density functional theory (DFT) calculations. We also performed a theoretical screening of six other ESIPT molecules, all of which could potentially undergo iEIPT upon electron attachment. More importantly, why ESIPT and iEIPT should occur to the same category of molecules are discussed by studying their local electronic properties. With the examples and evidences provided in the current study, we shed light on a huge list of iEIPT molecules, which promise to have applications such as molecular electronic devices that are responsive to electrons or current.

## Results

### Photoelectron spectroscopy

**Fig. 1:**
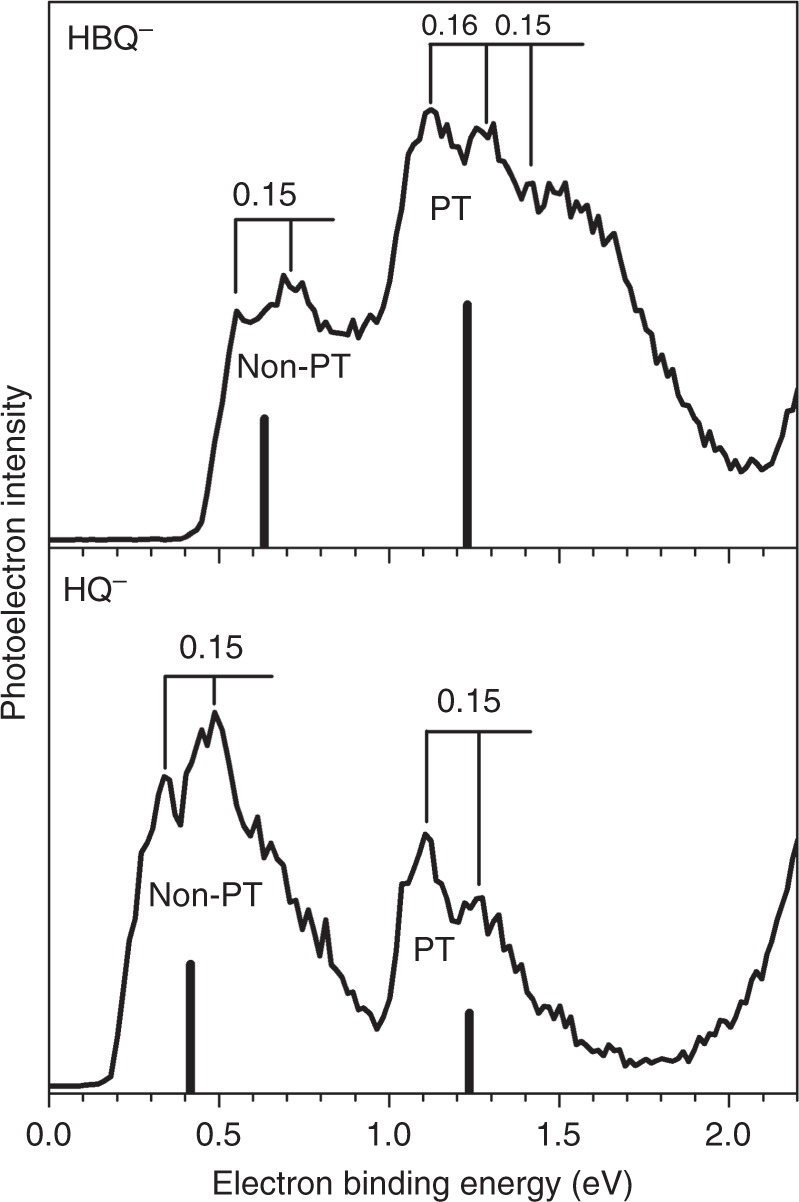
Photoelectron spectroscopy. Photoelectron spectra of HBQ^−^ and HQ^−^ taken with a 532 nm laser. PT proton-transferred, Non-PT non-proton-transferred

Figure 1 presents the photoelectron spectra of HBQ^−^ and HQ^−^ taken with 532 nm laser (2.33 eV). Both of the spectra possess two electron binding energy (EBE) bands, they corresponding to the non-proton-transferred (Non-PT) and the proton-transferred (PT) isomers. All of the EBE bands in these two spectra exhibit vibrational progressions of ~0.15 eV, which are attributable to the transitions from the anion’s ground state to different vibrational states of the neutral isomers (vide infra). For the HBQ^−^ spectrum, the Non-PT band starts from 0.45 eV and peaks at 0.68 eV, and the PT band starts from around 0.85 eV (value obtained by extrapolating the left EBE side) and peaks at 1.26 eV. Therefore, the experimental vertical detachment energies (VDE) of the Non-PT and PT HBQ^−^ isomers, i.e. the peak values of each EBE band, are 0.68 and 1.26 eV, respectively. For the HQ^−^ spectrum, the Non-PT band starts from 0.2 eV and peaks at 0.48 eV, and the PT band starts from 0.95 eV (by extrapolation) and peaks at 1.12 eV. Hence, the experimental VDE values of the Non-PT and PT HQ^−^ isomers are 0.48 and 1.12 eV, respectively. All of these VDE values are tabulated in Table 1 for comparison with the calculated results. Note that the PT band of HBQ^−^ is more intense than the Non-PT band, and HQ^−^ shows the opposite. The PT band is also broader than the Non-PT band for HBQ^−^ but those of HQ^−^ look similar; these observations will be explained in the following discussions. The electron affinity (EA) of a molecule is defined as the energy difference between the ground state of the anion and the ground state of the neutral. Only if when there is enough Franck−Condon overlap between these two states can EA be experimentally observed. In regard of this, the EAs of both HBQ and HQ cannot be observed in the spectra, and this statement will be discussed with the calculated potential energy surfaces in the following paragraphs. The adiabatic detachment energy (ADE) of an anion is defined as the energy difference between a certain anionic isomer and the neutral relaxed to the anion’s nearest local minima. In the current specific case, the ADE of the Non-PT anion is the energy difference between the Non-PT anion and the Non-PT neutral, and the ADE of the PT anion is the energy difference between the PT anion and the PT neutral, if there is a PT neutral at all.

**Table 1:** Experimental and theoretical vertical detachment energies (VDE) and adiabatic detachment energies (ADE) for non-proton-transferred and proton-transferred 10-hydroxybenzo[h]quinoline anions, non-proton-transferred and proton-transferred 8-hydroxyquinoline anions. Calculated electron affinities (EA) for both neutral systems are also presented. All numbers are in eV

		Experimental VDE	Theoretical VDE	Expt. ADE	Theo. ADE	Theo. EA
HBQ^−1/0^	Non-PT HBQ^−^	0.68	0.634	0.45	0.377	0.463
	PT HBQ^−^	1.26	1.231	NA	0.463	
HQ^−1/0^	Non-PT HQ^−^	0.48	0.417	0.2	0.177	0.253
	PT HQ^−^	1.12	1.236	0.95	0.997	

### Theoretical results

**Fig. 2:**
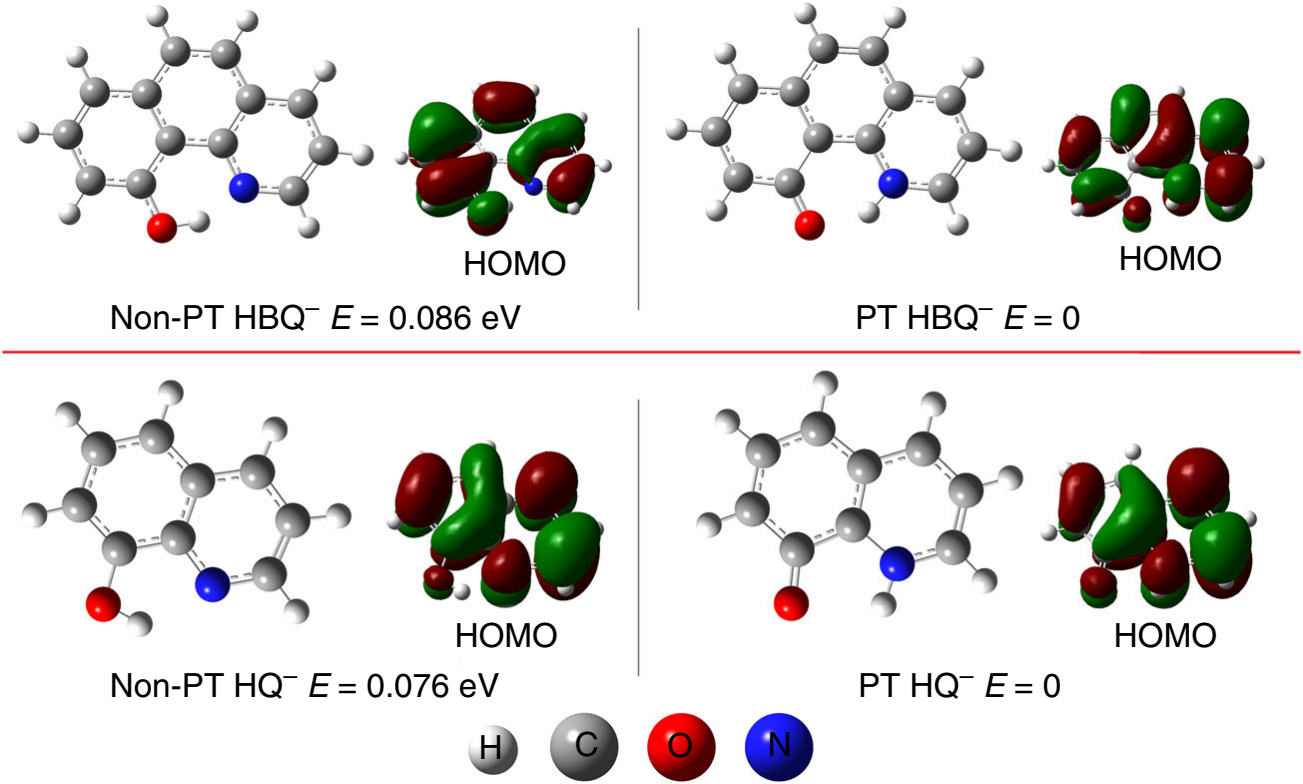
Calculated structures. Calculated structures of PT and Non-PT HBQ^−^ and HQ^−^, with their relative energies and HOMOs listed alongside. PT proton-transferred; Non-PT non-proton-transferred

Figure 2 shows the structures of the Non-PT and PT HBQ^−^ and HQ^−^ anions with their corresponding highest occupied molecular orbitals (HOMO, where the excess electron dwells) displayed aside. The relative energies corrected by the zero-point energies (ZPE) are also presented. The 3D coordinates of all the species calculated are provided in Supplementary Table 1. For both HBQ^−^ and HQ^−^, the PT isomers have lower energy than the Non-PT ones, suggesting that proton transfer is thermodynamically favored. The singly occupied HOMOs of all these four systems are delocalized π* antibonding orbitals, which satisfy the third criterion of searching for iEIPT molecules and also explain the stability of the anions. Both HBQ and HQ undoubtedly fulfill the first and second criteria based on their geometric nature.

**Fig. 3:**
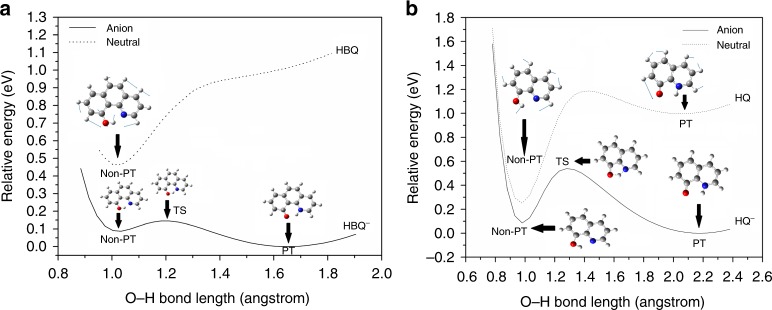
Potential energy surfaces. Potential energy surfaces of HBQ^0/−^ (**a**) and HQ^0/−^ (**b**) along the O−H coordinate using the relaxed scan method. PT proton-transferred, Non-PT non-proton-transferred, TS transition state

Figure 3 exhibits the potential energy surfaces of neutral and anionic HBQ (Fig. 3a) and HQ (Fig. 3b) along the O−H bond using the relaxed scan method. For each scan step, only the O−H bond distance is fixed and the rest of the molecule relaxes. The neutral potential energy surface is given by a dashed line and the anion by a solid line. For HBQ, the neutral only has one potential energy well, corresponding to the Non-PT neutral HBQ. The PT neutral does not exist. This observation confirms that the proton transfer in the anion was indeed induced by the excess electron since the neutral does not naturally have the PT isomer, or alternatively stated, EIPT did occur. For the anion potential energy surface, two wells were discovered, corresponding to the Non-PT and PT isomers. The low barrier between the two wells, 0.06 eV, together with the fact that PT isomer is thermodynamically favored compared to the Non-PT isomer, justify the observation of the more intense PT band. Here we conclude the mechanism of forming the PT HBQ^−^ as follows: Non-PT neutral HBQ obtains an electron, giving the Non-PT HBQ^−^, which partially undergoes iEIPT to yield the PT HBQ^−^. The anionic and neutral HBQ structures corresponding to each well are also embedded in Fig. 3a. The transition state (TS) between the Non-PT and PT isomers displays a six-member ring with the proton in the middle of the oxygen and nitrogen atoms. For the neutral molecule, we also present the calculated vibrational mode with the displacement vectors; this motion is the C−H and O−H shear mode, calculated to be 1319 cm^−1^, close to the experimental value, 0.15 eV. The experimental ADE of the Non-PT anion is the energy difference between the Non-PT anion and Non-PT neutral, corresponding to the onset of the Non-PT band in the photoelectron spectrum, 0.45 eV, in good consistency with the single point calculation value with ZPE correction (0.377 eV). The ADE of PT HBQ^−^, however, cannot be observed by experiment, because there is not a neutral potential well above the PT HBQ^−^, and the nearest neutral minimum is still the Non-PT neutral. So the ADE of the PT HBQ^−^ should be the energy difference between the Non-PT neutral and the PT anion. Due to the lack of Franck−Condon overlap, the extrapolated onset of the PT band (0.85 eV) in the spectrum is much higher than the calculated ADE (0.463 eV). The lack of Franck−Condon overlap also explains why the PT band is much wider than the Non-PT band in the spectrum. According to Fig. 3a, the EA of HBQ should be the energy difference between the PT anion and the Non-PT neutral, this value being the same as the ADE of the PT anion, which cannot be observed by experiment, neither, so we only report the theoretical EA result, 0.463 eV, in Table 1.

Figure 3b shows the potential energy surfaces of neutral and anionic HQ along the O−H bond, where both the anion and the neutral have two wells, corresponding to Non-PT and PT isomers, respectively. The existence of the PT isomer of the neutral gives rise to the experimental observation of the ADE of the PT HQ^−^ (extrapolated to be 0.95 eV), in good agreement with the calculated result (0.997 eV). The experimental ADE of the Non-PT HQ^−^, 0.2 eV, also agrees well with the calculated 0.177 eV. The photodetachment from PT HQ^−^ to PT HQ has a very good Franck−Condon overlap, resulting in a narrower PT band in the photoelectron spectrum compared to the PT HBQ^−^ case. The barrier between Non-PT and PT HQ^−^, 0.45 eV, is much higher than that of HBQ^−^ (0.06 eV); this is because the five-member ring TS of HQ^−^ has more tension than a six-member ring. This higher barrier also explains the relative lower intensity of PT HQ^−^ band compared to the Non-PT HQ^−^ band in the spectrum. Due to this relatively higher barrier, the PT isomer should increase with increased reaction time. With a longer delay between the photoemission laser in the ion source and the ion extraction voltage, the Non-PT isomer is given a longer time for the proton to transfer. Accordingly, the PT isomer’s intensity increases with the longer delay (Supplementary Figure 1). A similar observation was made in our previous studies^39^. The EA of HQ should be the energy difference between PT HQ^−^ and Non-PT HQ, again, due to insufficient Franck−Condon overlap, we only report the calculated EA value, 0.253 eV, in Table 1. For the neutral HQ, the PT isomer is 0.74 eV higher in energy compared to the Non-PT isomer, which makes HQ mostly Non-PT at room temperature. Hence, the mechanism of iEIPT of HQ is similar to that of HBQ: Non-PT HQ obtains an electron, giving the Non-PT HQ^−^, which further undergoes iEIPT to yield the PT HQ^−^. Again, we present the displacement vectors of the experimentally observed vibrational progressions for both Non-PT and PT neutrals. Calculated frequencies of the Non-PT (1265 cm^−1^) and PT (1191 cm^−1^) neutrals agree well with the experimental values (~0.15 eV).

In biomedia, an environment full of water, the potential energy surfaces of the anionic species in Fig. 3 will be significantly lowered compared to their neutral counterparts due to stronger hydrogen bonding with the negative charge. Therefore, solvation stabilizes these anionic species, which often results in blue shifts in the photoelectron spectra (see e.g. ref. ^3^), making these anionic species more likely to be present in vivo.

To sum, due to the coexistence of the Non-PT and PT anionic isomers and the fact that neutral molecules naturally exist in the Non-PT form, it can be concluded that the stepwise mechanism of iEIPT is: Non-PT neutral ${\rightarrow}$ Non-PT anion ${\rightarrow}$ PT anion.

## Discussion

**Fig. 4:**
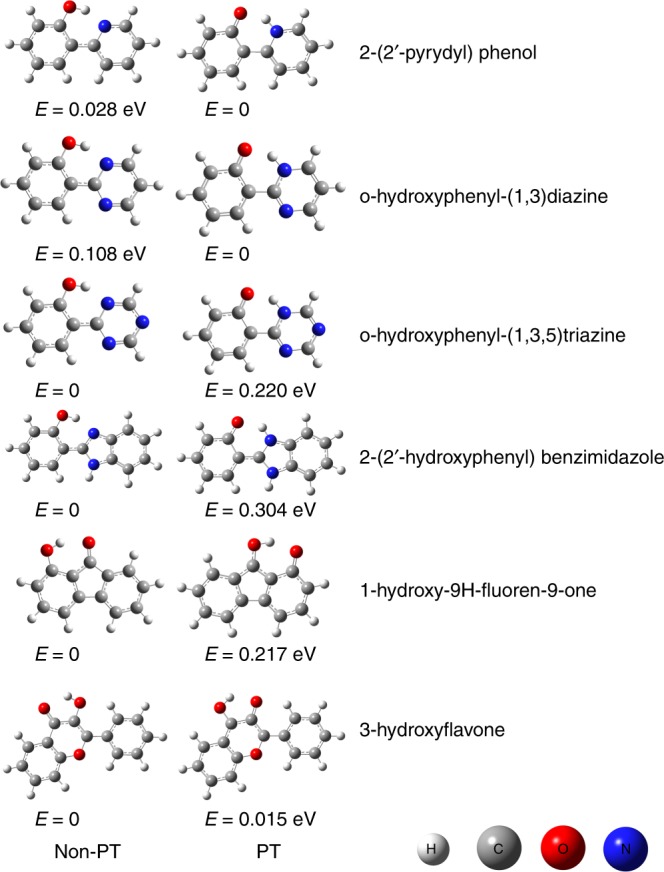
Molecular anion structures and relative energies. Structures and relative energies of the PT and Non-PT forms of several molecular anions. PT proton-transferred, Non-PT non-proton-transferred

Experimental and theoretical evidences have been provided in the current study to reveal the iEIPT capability of HBQ and HQ. This work also aims to provide evidences for the correlation between iEIPT and ESIPT. Here the iEIPT potential of six other molecules is screened at the ωB97XD/6–31 + G(d,p) level of theory, they being 2-(2′-pyrydyl) phenol, o-hydroxyphenyl-(1,3)diazine, o-hydroxyphenyl-(1,3,5)triazine, 2-(2′-hydroxyphenyl) benzimidazole, 1-hydroxy-9H-fluoren-9-one and 3-hydroxyflavone, all of which are also ESIPT molecules^24,36,40–42^. Figure 4 presents the PT and Non-PT anionic isomers and their relative energies of these six molecules. For 2-(2′-pyrydyl) phenol and o-hydroxyphenyl-(1, 3) diazine, the PT isomers have lower energy than the Non-PT ones, indicating that EIPT is favored energetically. For o-hydroxyphenyl-(1,3,5) triazine, 2-(2′-hydroxyphenyl) benzimidazole, 1-hydroxy-9H-fluoren-9-one and 3-hydroxyflavone, the PT isomers have slightly higher energy than the Non-PT ones. Evidenced by HQ and HBQ, the relative higher or lower energy of PT and Non-PT isomers does not necessarily eliminate a certain isomer’s existence, and they should by and large coexist upon receiving the excess electron.

**Fig. 5:**
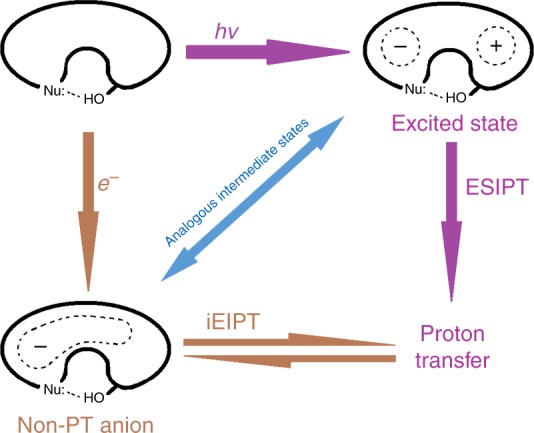
Proposed mechanism. Mechanisms and correlation of excited-state intramolecular proton transfer (ESIPT) and intramolecular electron-induced proton transfer (iEIPT). Nu nucleophile, Non-PT non-proton-transferred

Next we discuss the obvious question: apart from the three basic criteria, why do ESIPT and EIPT occur to the same group of molecules? In Fig. 5, we brief the mechanism of ESIPT along the purple arrows, the mechanism of iEIPT along the brown arrows, and the molecule of interest is simplified as an anomalous circle to denote the body of a π-conjugate system, with a Nu: to denote the proton receptor and an –OH group to denote the proton donor. Robb and his coworkers^40^, as well as Sobolewski and Domcke^43^ separately performed detailed computational studies of the ESIPT mechanism. They all pointed out that the transient excited state of the neutral, key to the proton transfer, is a charge-transfer state, where the proton receptor part of the molecule is negatively charged and the donor part is positively charged. Hence, the proton’s automatic transfer to the negatively charged site of the molecule is driven by the charge separation. In the iEIPT case along the brown arrows, upon electron attachment, the negative charge is delocalized on the ring of the Non-PT isomer; however, it is more localized on the more electronegative Nu: side. In the current case, Nu: is the nitrogen atom, and it draws −0.63 *e* in Non-PT HBQ^−^ and −0.67 *e* in Non-PT HQ^−^ as revealed by the natural population analysis (NPA). The NPA charges of the proton receptor sites of those species in Fig. 4 are tabulated in Supplementary Table 2, all of which exhibit high negative charge (from −0.5 to −0.8 *e*). The Non-PT anionic state can be viewed as an intermediate state towards PT, which has the unevenly distributed negative charge and “stimulates” the transfer of the proton to the more negatively charged side of the ring, i.e. the Nu:. Apparently, the Non-PT anionic state in EIPT is analogous to the excited state in ESIPT, explaining why these two rather distinct processes should occur to the same group of molecules. Remarkably, Sobolewski and Domcke also opined that in the intermolecular excited-state proton transfer processes, the charge transfer of the transient excited state was again the driving force of proton transfer^43^, which is also analogous to the intermolecular EIPT examples we previously studied^1–3,6–19^.

To conclude, in this work we provide evidence for the correlation between ESIPT and iEIPT molecules: if a molecule could undergo ESIPT, it could well be able to undergo iEIPT. Experimental and theoretical evidences are given to support the iEIPT properties of two ESIPT molecules, HBQ and HQ. Theoretical calculations investigate the viability of iEIPT for six other ESIPT molecules. This remarkable correlation between iEIPT and ESIPT processes arises from their similar states right before the proton transfer step, where the proton receptor side of the molecule is more negatively charged; as a result, the proton transfers. The discovery of the long list of iEIPT molecules could potentially have the applications as molecular devices (e.g. molecular switch) that are sensitive to stimuli such as electrons or current.

## Methods

### Experimental methods

Anion photoelectron spectroscopy is conducted by crossing a mass-selected beam of negative ions with a fixed-frequency photon beam and energy-analyzing the resultant photodetached electrons. It is governed by the energy-conserving relationship, *hν* = EBE + EKE, where *hν* is the photon energy, EBE is the electron binding (transition) energy, and EKE is the electron kinetic energy. Our anion photoelectron spectrometer, which has been described previously^44^, consists of a laser vaporization anion source, a linear time-of-flight mass analyzer/selector, a pulsed Nd:YAG photodetachment laser, and a magnetic bottle electron energy analyzer. Photoelectron spectra were calibrated against the well-known photoelectron spectrum of Cu^−^^45^. Both HBQ and HQ were purchased from Alfa Aesar® and used without further purification. Parent anions of HBQ and HQ were generated in a photoemission ion source. Briefly, a copper rod was interrogated by a pulsed Nd:YAG laser beam operating at a wavelength of 532 nm to photoemit electrons. HBQ or HQ was slightly heated to 40 °C in an oven placed in between the photoemission hosing and a pulsed valve, which supersonically expands a plume of ultrahigh purity helium gas (backing pressure 100 psi) to cool and carry the plasma. Negatively charged anions were then extracted into the spectrometer prior to mass selection and photodetachment. The delay between the photoemission laser in the ion source and the ion extraction voltage is varied to allow different proton transfer time periods in order to distinguish between isomers.

### Theoretical methods

DFT calculations were conducted by applying the hybrid meta-GGA functional ωB97XD using the Gaussian09 software package to determine the geometries of both neutral and anionic isomers HBQ and HQ, the adiabatic electron affinities (EA), the ADE, the VDE and the potential energy surface along the O–H bond. All geometries, including that of the anion and its corresponding neutral molecule, were fully optimized without any geometrical constraints using the 6–31++G (d, p) basis set and then improved by single point energy calculations with a larger basis set 6–311++G (3df, 3pd). EA is calculated for the neutral molecules. ADE and VDE were calculated for both the proton-transferred and non-proton-transferred anionic isomers. The potential energy surface was scanned along the O−H coordinate with a step width of 0.05 Å by sequentially relaxing the rest of the molecule to its ground state to reveal the barrier height and potential wells along the proton transfer pathway. Natural population analysis was utilized to reveal the atomic charges in the species of interest.

## Supplementary information

Supplementary Information

## Data Availability

The authors declare that the data supporting the findings of this study are available within the paper and its supplementary information files, and from the authors upon reasonable request.
