# Supplementary material for: Intramolecular electron-induced proton transfer and its correlation with excited-state intramolecular proton transfer
Source: Nat Commun. 2019 Mar 12;10:1170. doi: 10.1038/s41467-019-09154-5 (PMC6414547; doi:10.1038/s41467-019-09154-5)
Supplement: Supplementary file 1 — Supplementary Information [file 41467_2019_9154_MOESM1_ESM.pdf]

Supplementary Information

Intramolecular Electron-Induced Proton Transfer and its Correlation with Excited-State Intramolecular Proton Transfer

Wang et al.

Supplementary Table 1. Calculated 3D coordinates of all the species in the main text.

Non-PT HBQ$^-$			
C	3.54756900	-0.37035500	0.00001200
C	2.97821800	0.91292700	0.00001500
C	1.55056400	1.03342700	-0.00000600
C	0.78685400	-0.17461400	-0.00004800
C	2.73486500	-1.48434500	0.00000800
C	0.86727400	2.26852300	0.00000600
C	-0.65580100	-0.09879100	-0.00001200
C	-1.30648700	1.17712200	-0.00000300
C	-0.51695900	2.34811600	-0.00000300
C	-2.73111900	1.22948500	0.00000400
H	-3.21906300	2.20151900	0.00000300
C	-3.47081100	0.07173500	0.00000700
C	-2.84795600	-1.19350100	0.00000600
C	-1.46269400	-1.28587200	0.00000400
H	1.45979200	3.18248000	0.00002200
H	4.62802500	-0.49698900	0.00002900
H	3.59522600	1.80760900	0.00002300
H	3.14793800	-2.48964000	0.00001700
H	-1.01183200	3.31666900	0.00000600
H	-4.55789500	0.12585800	0.00000700
H	-3.42764600	-2.11117900	0.00001100
N	1.36796500	-1.40059700	-0.00002600
O	-0.89353500	-2.50033100	0.00001300
H	0.11687400	-2.33264300	0.00001400
PT HBQ$^-$			
C	-3.55260200	-0.35793100	0.00001200
C	-2.96880600	0.91938800	-0.00001000
C	-1.53368800	1.03490400	-0.00000700
C	-0.75426000	-0.14223300	0.00000100
C	-2.76464900	-1.48404900	0.00003100
C	-0.84812900	2.27413500	-0.00000500
C	0.67216800	-0.11099500	-0.00000200
C	1.32340300	1.16145000	0.00000900
C	0.52405500	2.33990700	0.00000600
C	2.73873000	1.22995200	0.00001700
H	3.22652100	2.20152000	0.00002700
C	3.47444700	0.05773100	0.00001200
C	2.86535100	-1.19978000	-0.00001100
C	1.45662300	-1.35231300	-0.00002800
H	-1.43717700	3.18990300	-0.00000800
H	-4.63302500	-0.47315800	0.00002600
H	-3.57937900	1.81741300	-0.00001400
H	-3.15677500	-2.49344000	0.00005600
H	1.02493500	3.30569100	0.00000900
H	4.56283700	0.11384100	0.00001800
H	3.45956500	-2.10949500	-0.00002200
N	-1.39110400	-1.36454600	0.00001400
O	0.89816300	-2.50846900	-0.00005000

H	-0.71093800	-2.15369500	0.00006200
Non-PT HQ^-			
C	2.35532800	-0.06409400	-0.00006700
C	1.29027000	0.83321200	-0.00003200
C	-0.05216800	0.39766900	0.00002400
C	-0.33220700	-1.00262400	0.00003500
C	0.76216400	-1.90097100	0.00000600
C	2.07678600	-1.43576300	-0.00002100
H	3.37438700	0.31210000	-0.00011000
C	-1.70674000	-1.38330100	0.00006500
H	0.56197800	-2.97107800	0.00002000
H	2.89892100	-2.14863200	-0.00004600
C	-2.68303300	-0.35743700	0.00001600
C	-2.29910900	0.97077400	-0.00011000
H	-1.98336700	-2.43449200	0.00012400
H	-3.74315900	-0.60324000	0.00005000
H	-3.05220900	1.75848100	-0.00011900
O	1.49198600	2.18516400	0.00004600
N	-1.00510000	1.38962500	-0.00007300
H	0.57551800	2.53339000	0.00072300
PT HQ^-			
C	2.37795500	-0.01427200	0.00000400
C	1.32883900	0.94917800	-0.00002400
C	0.00311800	0.37949800	-0.00004900
C	-0.28032400	-0.99605100	-0.00002700
C	0.81134900	-1.89376100	-0.00001500
C	2.10905600	-1.38539100	0.00000000
H	3.40192400	0.35173400	0.00003600
C	-1.66907200	-1.39802800	-0.00000100
H	0.62759900	-2.96556000	-0.00000200
H	2.94661800	-2.08300500	0.00001800
C	-2.67079300	-0.41201000	0.00006800
C	-2.35418100	0.93008100	0.00004600
H	-1.92348000	-2.45391400	0.00002800
H	-3.72049200	-0.69442700	0.00013900
H	-3.09329300	1.72212100	0.00007400
O	1.46982500	2.21977700	0.00003800
N	-1.03284200	1.30626700	-0.00008900
H	-0.70326000	2.26550700	0.00001300
Non-PT 2-(2’-pyrydyl) phenol			
C	-2.81036900	0.89907100	0.00053800
C	-1.42474900	0.95500800	0.00089400
C	-0.62931600	-0.25059300	-0.00006900
C	-1.37004300	-1.46944500	-0.00091600
C	-2.76031600	-1.50771700	-0.00087200
C	-3.50076500	-0.32554200	-0.00037700
H	-3.34630800	1.84482600	0.00101600
H	-0.83473100	-2.41375100	-0.00176200
H	-3.26519900	-2.47167200	-0.00158700
H	-4.58744900	-0.34355300	-0.00060000

C	0.80438900	-0.21455600	0.00009100
C	1.63265100	-1.38763600	0.00147500
C	2.99822100	-1.28662600	0.00109800
H	1.17583900	-2.37171800	0.00301800
C	2.76179700	1.09659200	-0.00134600
C	3.61701800	-0.00060400	-0.00057700
H	3.60624500	-2.18925800	0.00216100
H	3.17799600	2.10590800	-0.00245400
H	4.69411900	0.12687500	-0.00103900
N	1.43653300	1.03281000	-0.00103900
O	-0.84229300	2.17792500	0.00110900
H	0.15099300	2.01156300	0.00001000
PT 2-(2'-pyrydyl) phenol			
C	-2.83373100	0.90862600	0.00304200
C	-1.40464700	1.01120000	0.00172200
C	-0.67335200	-0.24566500	-0.00081600
C	-1.39714300	-1.44858600	-0.00419300
C	-2.78548200	-1.50482300	-0.00346500
C	-3.50296800	-0.30007200	0.00055900
H	-3.37749200	1.85013200	0.00587000
H	-0.85306900	-2.38975300	-0.00804200
H	-3.29659500	-2.46367500	-0.00632200
H	-4.59218600	-0.31405100	0.00133400
C	0.80142700	-0.26648500	-0.00027500
C	1.60855300	-1.38456400	0.00496700
C	3.02593500	-1.29101400	0.00435300
H	1.15041600	-2.36722400	0.01039300
C	2.80090000	1.10954000	-0.00444300
C	3.60712700	0.00947200	-0.00011400
H	3.64162100	-2.18380100	0.00877300
H	3.18150800	2.12487100	-0.00736400
H	4.68532400	0.14359400	0.00045300
N	1.42662200	0.98375700	-0.00605300
O	-0.84442000	2.16927700	0.00413400
H	0.74976600	1.77362800	-0.00382600
Non-PT o-hydroxyphenyl-(1,3) diazine			
C	-2.83953900	-1.30176400	-0.00003000
C	-0.82184900	-0.18770700	-0.00004600
C	-2.77778600	1.07143600	0.00005500
H	-3.37583900	-2.25447200	-0.00007600
H	-3.24103000	2.05977900	0.00008700
C	0.60674500	-0.22774400	-0.00001900
C	1.41858700	0.96073600	-0.00003700
C	1.30723800	-1.46695100	0.00002300
C	2.80260900	0.87588700	-0.00001200
C	2.69471300	-1.53390000	0.00004500
H	0.71205700	-2.37383200	0.00003400
C	3.46080000	-0.36512100	0.00003100
H	3.36193200	1.80807300	-0.00002200
H	3.18231600	-2.50668000	0.00007700

H	4.54700400	-0.40774600	0.00004900
N	-1.46312800	1.04981300	0.00001700
N	-1.53261800	-1.38193400	-0.00007300
O	0.85817000	2.19812300	-0.00003300
H	-0.12834100	2.05665500	0.00003600
C	-3.56846900	-0.08895500	0.00005400
H	-4.65153400	-0.05740800	0.00008100
PT o-hydroxyphenyl-(1,3) diazine			
C	-2.87757700	-1.29829600	0.00003400
C	-0.82091400	-0.24933700	-0.00027000
C	-2.81935900	1.09293500	-0.00008300
H	-3.40926000	-2.24450900	0.00016100
H	-3.23340000	2.09200600	-0.00008100
C	0.65316800	-0.22600700	-0.00005500
C	1.38845900	1.02078400	0.00037400
C	1.33842600	-1.44964900	-0.00013800
C	2.81914400	0.89615100	0.00027400
C	2.72215500	-1.52534600	-0.00005900
H	0.73436600	-2.35265700	-0.00032800
C	3.45748900	-0.32597200	0.00010200
H	3.38304400	1.82562300	0.00039200
H	3.22500600	-2.48833300	-0.00017500
H	4.54657300	-0.36031400	0.00013500
N	-1.42854600	0.97924400	-0.00053400
N	-1.48943600	-1.37867400	-0.00006900
O	0.83788100	2.18085000	0.00028200
H	-0.74975500	1.77129700	-0.00045200
C	-3.55052300	-0.07733400	0.00014300
H	-4.63656200	-0.04148100	0.00038600
Non-PT o-hydroxyphenyl-(1,3,5)triazine			
C	-2.85192900	-1.23704300	0.00004700
C	-0.82358000	-0.18912500	-0.00003600
C	-2.79152800	1.00661300	-0.00001900
H	-3.43480600	-2.16030600	0.00001700
H	-3.30076200	1.97145000	0.00011400
C	0.60231800	-0.22890900	-0.00003000
C	1.40900000	0.96101900	0.00000300
C	1.29989000	-1.46773900	-0.00004200
C	2.79387400	0.87697700	0.00005800
C	2.68688100	-1.53392100	0.00000000
H	0.70361500	-2.37405400	-0.00008200
C	3.44984600	-0.36290100	0.00005800
H	3.35353700	1.80865300	0.00009000
H	3.17645700	-2.50524200	-0.00000800
H	4.53594200	-0.40478400	0.00010500
N	-1.48191900	1.03851700	-0.00005300
N	-1.55423000	-1.36966400	-0.00006500
N	-3.58274900	-0.08680700	0.00010800
O	0.84828800	2.19703500	-0.00004200
H	-0.13661600	2.06385300	-0.00007100

PT o-hydroxyphenyl-(1,3,5)triazine			
C	-2.88863200	-1.23709200	-0.00049400
C	-0.81960500	-0.25350200	0.00057800
C	-2.83676800	1.02148700	-0.00012100
H	-3.46637000	-2.15425000	-0.00120300
H	-3.30065400	2.00047400	-0.00032700
C	0.64585500	-0.22819900	0.00031200
C	1.37588300	1.02229700	-0.00025000
C	1.33360100	-1.45279000	0.00037200
C	2.80836900	0.90029600	-0.00047300
C	2.71530500	-1.52359600	0.00006500
H	0.73138000	-2.35723200	0.00067900
C	3.44736800	-0.31984500	-0.00032800
H	3.37060400	1.83056500	-0.00078700
H	3.22214600	-2.48420800	0.00018700
H	4.53633700	-0.35217800	-0.00056200
N	-1.44450400	0.97384400	0.00206600
N	-1.50700500	-1.36833800	-0.00033600
N	-3.55954200	-0.07226200	-0.00108000
O	0.82268200	2.17863300	-0.00021000
H	-0.78579300	1.78072200	0.00117500
Non-PT 2-(2’-hydroxyphenyl) benzimidazole			
C	2.00576700	-0.80230900	-0.00011800
C	1.89089000	0.61904000	-0.00284400
C	3.06326100	1.38920200	-0.00249600
C	4.30035900	0.73123600	0.00102800
C	4.39216300	-0.66133200	0.00415600
C	3.22641300	-1.45485500	0.00343800
C	-0.15636600	-0.15298400	-0.00304500
H	3.00041500	2.47312700	-0.00450100
H	5.21368300	1.32206800	0.00152500
H	5.36641000	-1.14220000	0.00713400
H	3.28941200	-2.54021100	0.00558900
N	0.58314600	0.99325000	-0.00486700
C	-1.56070100	-0.23455700	-0.00204000
C	-2.35810700	0.96911300	0.00102900
C	-2.27625000	-1.47584100	-0.00309900
C	-3.73624200	0.90361500	0.00382300
C	-3.66598700	-1.51710700	-0.00021900
H	-1.72704700	-2.41501400	-0.00748200
C	-4.41428700	-0.33913200	0.00346000
H	-4.28587400	1.84106700	0.00635000
H	-4.16867000	-2.48217800	-0.00126100
H	-5.50020400	-0.36844300	0.00575800
O	-1.76297900	2.18769700	0.00159800
H	-0.78088500	2.05001400	-0.00078700
N	0.70467600	-1.25951000	-0.00300200
H	0.41635300	-2.22051100	0.01154600
PT 2-(2’-hydroxyphenyl) benzimidazole			
C	2.00064400	-0.80931800	-0.00122500

C	1.89854800	0.60918800	-0.00368700
C	3.04828200	1.39781600	-0.00142300
C	4.29612200	0.74245400	0.00350100
C	4.39128900	-0.64213600	0.00638300
C	3.22857500	-1.44907500	0.00399900
C	-0.20561000	-0.22787800	-0.00530600
H	2.97806500	2.48029200	-0.00278200
H	5.20397000	1.34018200	0.00540300
H	5.36735900	-1.11867200	0.01059400
H	3.29906900	-2.53290100	0.00620000
N	0.57167100	0.91733600	-0.00735200
C	-1.60386000	-0.25592300	-0.00311000
C	-2.31642900	1.03206100	0.00082300
C	-2.34199200	-1.47009300	-0.00340600
C	-3.72961800	0.95690200	0.00566600
C	-3.73536800	-1.47494100	0.00128500
H	-1.81259300	-2.42323400	-0.00843300
C	-4.42527600	-0.25865200	0.00603100
H	-4.26582800	1.90290700	0.00910700
H	-4.27508900	-2.41904100	0.00087100
H	-5.51392100	-0.25311000	0.00986300
O	-1.67650700	2.14958800	0.00013300
H	0.01020200	1.79125000	-0.00511400
N	0.70638800	-1.29006100	-0.00641400
H	0.44257100	-2.25774400	0.01239400
Non-PT 1-hydroxy-9H-fluoren-9-one			
C	-3.47319500	-1.02587700	0.00008000
C	-3.66312300	0.37208900	0.00009700
C	-2.57687100	1.23699000	0.00009800
C	-1.27753400	0.71003200	0.00008100
C	-1.08370000	-0.71206300	0.00005900
C	-2.18786200	-1.56482500	0.00005900
H	-4.33651900	-1.68701400	0.00007800
H	-4.67396700	0.77396600	0.00011900
H	-2.71778200	2.31477300	0.00012700
H	-2.04849400	-2.64430100	0.00003300
C	0.98355800	0.29175000	-0.00002500
C	2.37893100	0.41602600	-0.00013000
C	3.15354500	-0.73785600	-0.00016300
C	2.51932200	-2.00056900	-0.00011500
C	1.13254700	-2.13429300	-0.00003700
C	0.34913900	-0.97207300	0.00001200
H	4.23675100	-0.65805000	-0.00023400
H	3.14021500	-2.89378300	-0.00015100
H	0.67655000	-3.12146900	-0.00001800
C	0.01847200	1.36883700	0.00012400
O	0.31403500	2.61085900	0.00012800
O	2.92618900	1.65719500	-0.00018800
H	2.16209000	2.28243900	-0.00030800
PT 1-hydroxy-9H-fluoren-9-one			

C	3.47156600	-1.02362300	-0.00011500
C	3.65640500	0.37064500	-0.00014800
C	2.56672300	1.23475100	-0.00011800
C	1.27171600	0.69879400	-0.00005600
C	1.07891800	-0.72813400	-0.00002300
C	2.18599600	-1.57048400	-0.00005300
H	4.33726300	-1.68128100	-0.00013700
H	4.66485200	0.77744000	-0.00019700
H	2.71146700	2.31164600	-0.00014200
H	2.05156200	-2.65001800	-0.00002800
C	-0.99445000	0.27414300	0.00003600
C	-2.41002400	0.48131700	0.00006600
C	-3.16963800	-0.73043500	0.00011200
C	-2.54152200	-1.97850600	0.00012000
C	-1.14281400	-2.14432800	0.00008000
C	-0.36009300	-0.99179100	0.00003600
H	-4.25453100	-0.65898300	0.00013800
H	-3.16769400	-2.86996700	0.00015300
H	-0.70392200	-3.13860900	0.00008200
C	-0.02757700	1.29680800	-0.00000400
O	-0.34451700	2.60014800	0.00001100
O	-2.88134400	1.67333900	0.00004200
H	-1.34335300	2.58692600	0.00011300
Non-PT 3-hydroxyflavone			
C	-0.05752400	1.40161800	0.00008100
C	-1.46959000	1.56726600	0.00007100
C	-2.25256900	0.34878400	0.00001000
C	-1.58016600	-0.89028100	-0.00002500
C	0.58093600	0.17005900	0.00005500
H	-4.15961000	1.30317500	-0.00000300
C	-3.65681500	0.34121800	-0.00002300
C	-2.28391800	-2.08961200	-0.00008500
C	-3.68359800	-2.07367500	-0.00011400
C	-4.36673300	-0.85769300	-0.00008500
H	-1.72803900	-3.02211300	-0.00010800
H	-4.22985300	-3.01295200	-0.00016200
H	-5.45365600	-0.84383700	-0.00011300
C	1.98145900	-0.13719500	0.00001300
C	2.42148300	-1.48815400	0.00024600
C	2.98842000	0.86580900	-0.00024800
C	3.77256300	-1.80646200	0.00021400
H	1.68217000	-2.28002000	0.00045100
C	4.33277900	0.52788500	-0.00027800
H	2.69385900	1.90727300	-0.00043100
C	4.75005200	-0.80868600	-0.00004800
H	4.06723500	-2.85384200	0.00039700
H	5.07373400	1.32417700	-0.00048200
H	5.80647400	-1.06188800	-0.00006800
O	-0.22579800	-0.97223400	0.00000700
O	0.61964500	2.58044000	0.00009800
H	-0.13068200	3.21947700	-0.00000600

O	-1.95413700	2.75370100	0.00012300
PT 3-hydroxyflavone			
C	-0.00640900	1.45312200	-0.00037500
C	-1.44020100	1.49696600	-0.00015400
C	-2.24245000	0.34510600	0.00004000
C	-1.56713000	-0.90371000	-0.00029700
C	0.59001800	0.17339900	-0.00048800
H	-4.18246000	1.27960700	0.00080600
C	-3.65893800	0.32924500	0.00052100
C	-2.26976500	-2.10142400	-0.00022900
C	-3.66964300	-2.09141400	0.00022700
C	-4.35528300	-0.87290500	0.00060800
H	-1.71036200	-3.03189100	-0.00052100
H	-4.21471000	-3.03087300	0.00029900
H	-5.44238600	-0.86308300	0.00100300
C	1.99133700	-0.14103700	-0.00019100
C	2.43981900	-1.48543100	-0.00023100
C	2.98096400	0.87531600	0.00017900
C	3.79506900	-1.79103300	0.00013000
H	1.70693700	-2.28398000	-0.00052500
C	4.33019400	0.55041500	0.00054000
H	2.64742100	1.90699100	0.00019600
C	4.75891800	-0.78120400	0.00052200
H	4.10265800	-2.83472600	0.00010900
H	5.06448900	1.35292900	0.00083000
H	5.81822100	-1.02374600	0.00083900
O	-0.21920000	-0.96661800	-0.00078300
O	0.59859800	2.58562400	-0.00032200
O	-1.96764900	2.74469500	0.00011900
H	-1.14280800	3.28669900	0.00003000

Supplementary Table 2. NPA negative charges (*e*) at the proton receptor sites of all the species in Figure 4 at the ωB97XD/6-31+G(d,p) level of theory.

Species	Proton receptor	Charge
2-(2'-pyrydyl) phenol	N atom	-0.60
o-hydroxyphenyl-(1,3)diazine	N atom	-0.59
o-hydroxyphenyl-(1,3,5)triazine	N atom	-0.62
2-(2'-hydroxyphenyl) benzimidazole	N atom	-0.64
1-hydroxy-9H-fluoren-9-one	O atom	-0.73
3-hydroxyflavone	O atom	-0.77

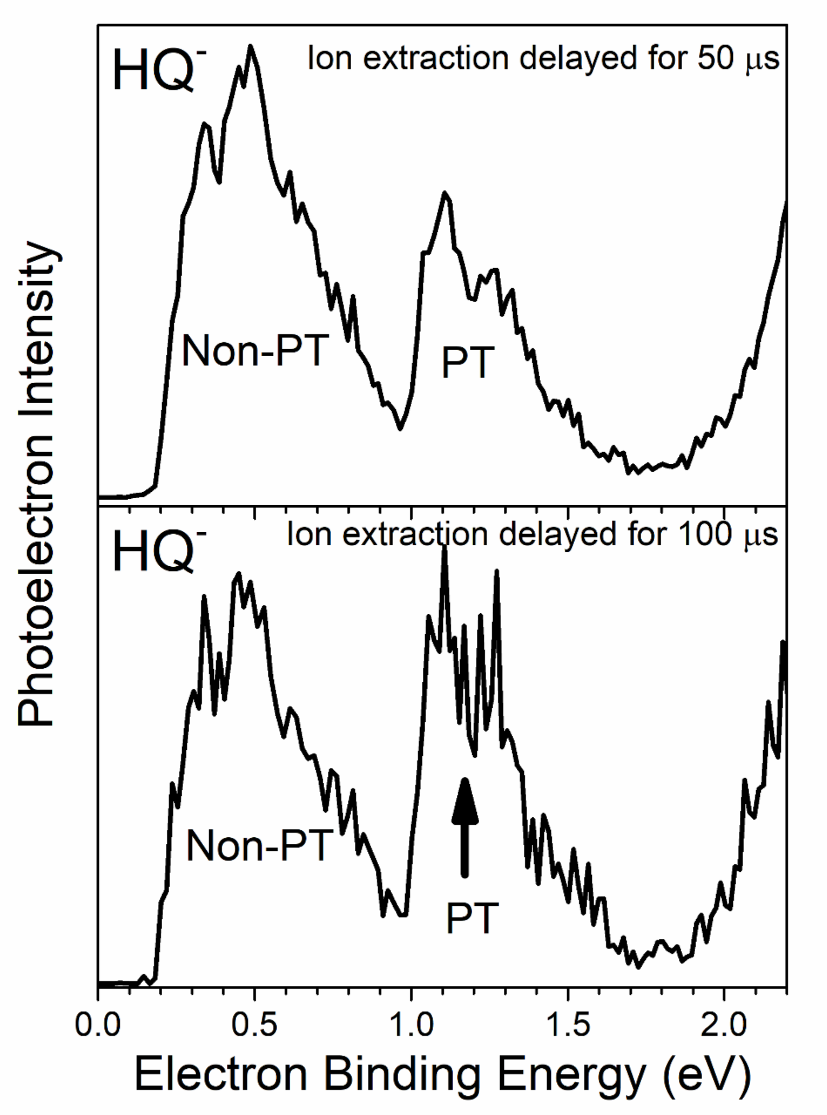

Supplementary Figure 1. The photoelectron intensity of PT HQ$^-$ increased with greater delay time between the laser trigger in the ion source and the ion extraction voltage.
